# Comparative Analysis of Pediatric Hospitalizations during Two Consecutive Influenza and Respiratory Virus Seasons Post-Pandemic

**DOI:** 10.3390/v15091825

**Published:** 2023-08-28

**Authors:** Santiago Presti, Sara Manti, Francesco Gambilonghi, Giuseppe Fabio Parisi, Maria Papale, Salvatore Leonardi

**Affiliations:** 1 Pediatric Respiratory Unit, AOUP “G. Rodolico-San Marco”, University of Catania, 95123 Catania, Italy; santiagopresti@gmail.com (S.P.); fgambilonghi@gmail.com (F.G.); giuseppeparisi88@hotmail.it (G.F.P.); mariellapap@yahoo.it (M.P.); leonardi@unict.it (S.L.); 2 Pediatric Unit, Department of Human Pathology in Adult and Developmental Age “Gaetano Barresi”, University of Messina, 98125 Messina, Italy

**Keywords:** COVID-19 pandemic, epidemiology, respiratory infections, respiratory infections, rhinovirus, RSV

## Abstract

Background: The COVID-19 pandemic has had a significant impact on the epidemiology of respiratory viruses. Non-pharmaceutical interventions (NPIs) led to a dramatic reduction in respiratory infections. However, the long-term effects on respiratory virus epidemiology remain unclear. Materials and Methods: We conducted a comparative study on hospitalized pediatric patients with respiratory illness during two seasons: 1 October 2021 to 15 March 2022 and 1 October 2022 to 15 March 2023. We compared the type of virus, mean duration of hospitalization, and disease severity. Results: In the first season, 47.1% of patients (65/138) tested positive for at least one respiratory virus, with respiratory syncytial virus (RSV) being the most frequent (23.2%). In the second season, 82.9% of patients (102/123) tested positive, with RSV and *Rhinovirus* being the most prevalent (28.38% and 27.03%, respectively). Other viruses, such as *Influenza* A/B, *Metapneumovirus*, and *Adenovirus*, also showed increased prevalence. Disease severity and mean duration of hospitalization were similar between the two seasons. Conclusions: Our study highlights increased prevalence in respiratory viruses, including RSV and *Rhinovirus*, following the easing of NPIs. The prevalence in respiratory viruses, including RSV and *Rhinovirus*, increased in the second season compared to the first one. Interestingly, RSV’s peak incidence shifted from February to November. The emergence of rhinovirus as the most prevalent respiratory virus during certain months suggests viral competition and dynamic changes in viral circulation. The overall severity of respiratory infections remained relatively stable between the seasons.

## 1. Introduction

The global experience of the severe acute respiratory syndrome coronavirus 2 (SARS-CoV-2) pandemic has significantly impacted the epidemiology of respiratory viruses. In 2020, implementing non-pharmaceutical interventions (NPIs), such as hand hygiene measures, social distancing, and face masks, led to a dramatic reduction in respiratory infections [1,2,3,4,5,6,7,8,9,10,11,12]. Several studies have reported substantial decreases in hospitalizations of children with respiratory conditions when comparing the Coronavirus Disease 2019 (COVID-19) outbreak with previous seasons affected by respiratory viruses [13,14,15]. In Italy, throughout 2020 and the beginning of 2021, numerous hospitals observed the absence of peak incidences in respiratory viruses and a significant decline in respiratory infections [3,4,6,16]. The COVID-19 pandemic has caused significant changes in respiratory virus infections worldwide. Reductions in respiratory virus infections, including influenza and the respiratory syncytial virus (RSV), were most pronounced during the initial phase of the COVID-19 pandemic and continued to varying degrees during subsequent waves of SARS-CoV-2 infections [17]. These reductions in community infection rates have resulted in fewer hospitalizations and deaths associated with non-SARS-CoV-2 respiratory infections [18]. In a previous study, we described the first epidemic of respiratory viruses in a pediatric cohort of 123 patients after the easing of NPIs for the containment of COVID-19, reporting new epidemiological trends in the 2021–2022 season [19]. In the first post-pandemic epidemic, a multicenter Italian study demonstrated an early onset of the epidemiological curve for RSV and revealed that SARS-CoV-2 exhibited competitive pressure on other respiratory viruses [20]. To monitor the epidemiological changes triggered by the pandemic, a new surveillance study until the last epidemic season has been conducted.

## 2. Materials and Methods

All children consecutively hospitalized due to respiratory illness at the Department of Clinical and Experimental Medicine, Pediatric Respiratory Unit, San Marco Hospital, University of Catania, were included in this study. The study period spanned from 1 October 2021 to 15 March 2022 and from 1 October 2022 to 15 March 2023. Respiratory virus testing was performed using a nasal swab on all included patients. The comparison between the two seasons focused on the following endpoints: viral agent, mean duration of hospitalization, and disease severity assessed by clinical respiratory score (CRS) [21]. The study was conducted in accordance with the principles outlined in the Declaration of Helsinki [22,23]. This retrospective study was approved by the local ethics committees (protocol code 18/2021, 18 January 2021). Patients with significant pre-existing conditions such as primary immunodeficiencies, oncological disorders, and cystic fibrosis were excluded. Consequently, the participants considered for this study were generally individuals without known chronic illnesses, with the exception of a respiratory virus infection. The patients included ranged in age from 1 month to 18 years. Given the complexity of co-infections and their potential to introduce confounding factors in disease severity and hospitalization, we chose to narrow our investigation to the primary virus of interest to maintain clarity and rigor in our analysis.

### Statistical Analysis

For statistical analysis, SPSS 28.0.1.1 software was used. A 2-tailed *t*-test for unpaired data was applied to compare the mean duration of hospitalization, and a chi-square test was applied to relate the severity of viruses.

## 3. Results

Over the period 1 October 2021 to 15 March 2022, among the 138 patients admitted to our department for respiratory illness, 47.1% (65/138 patients) resulted positive for at least one respiratory virus: RSV was the most frequent one (23.2%), followed by Bocavirus (10.87%), Rhinovirus (RV) (4.3%), Parainfluenza virus (2.9%), Enterovirus (2.17%), endemic Coronavirus (1.45%), and Metapneumovirus (1.45%). A total of 53.6% of patients were negative.

**Figure 1:**
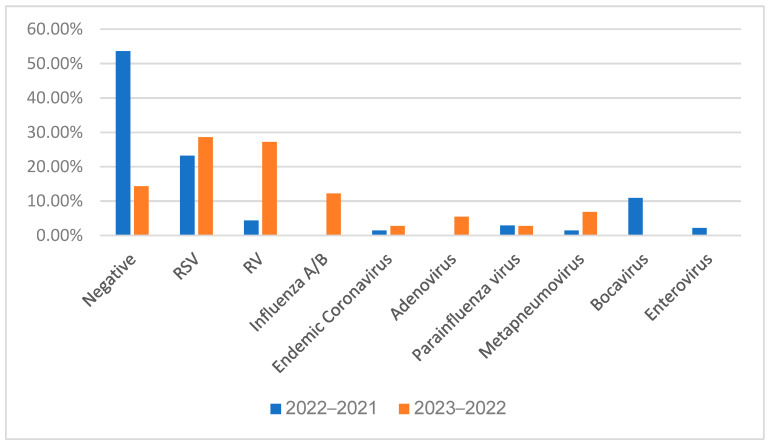
Viral prevalence through the waves 2021–2022 and 2022–2023. RSV: Respiratory syncytial virus; RV: Rhinovirus.

Over the period 1 October 2022 to 15 March 2023, among the 123 patients admitted to our department for respiratory illness, 102 resulted positive for at least one respiratory virus: RSV was the most frequent one (28.38%), followed by Rhinovirus (27.03%), Influenza A/B virus (12.16%), Metapneumovirus (6.76%), Adenovirus (5.41%), endemic Coronavirus (2.70%), and Parainfluenza virus (2.70%). No Bocaviruses were detected. A total of 14.29% of patients were negative. Figure 1 shows the differences between the two seasons.

**Figure 2:**
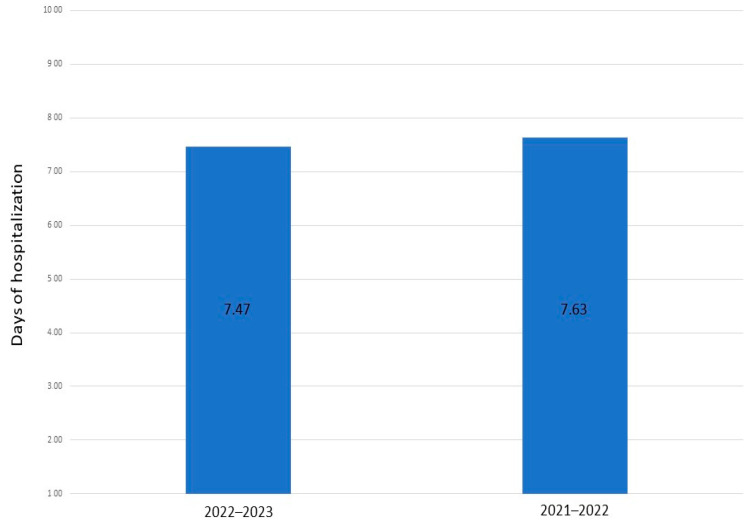
Mean duration of hospitalization through the waves 2021–2022 and 2022–2023.

In examining the severity, in 2021/2022, 105 patients had mild CRS values, 24 patients had moderate values, and 9 patients had severe values. In 2022/2023, 99 patients had mild CRS values, 15 patients had moderate values, and 9 patients had severe values. As shown in Figure 2, the mean duration of hospitalization was 7.63 ± SD 4.07 in 2021/2022 and 7.47 days ± SD 3.54 in 2022/2023.

## 4. Discussion

As suggested by the European Respiratory Society (ERS) in 2023, the emergence of SARS-CoV-2 has underscored the importance of respiratory surveillance for respiratory viruses, thereby shedding light on the lessons learned from the COVID-19 pandemic [24]. Respiratory viruses are a common cause of morbidity in children, alone or in synergy with bacterial pathogens [25]. Adopting containment measures for COVID-19 due to the pandemic has almost eliminated the circulation of endemic respiratory viruses and led to new possible epidemiological trends [3,4]. The lack of immune stimulation by viruses might have led to a drop in population immunity with consequent changes in respiratory virus epidemiology after the removal of NPIs [13,26]. Several studies have reported the recurrence of respiratory viruses out of season following the easing of containment measures. Specifically, a notable advancement in the peak incidence in RSV and other respiratory viruses has been observed [9,27,28,29]. The lack of immune stimulation might lead to higher hospitalization rates in children due to RSV, as reported by Rao et al. who highlighted an increase in hospital admissions for RSV among older children compared to previous seasons [30]. Other centers have also documented an unusually higher prevalence in non-RSV respiratory viruses. Tempte et al. reported a resurgence in *Influenza* virus in a community among kindergarteners from September to December [31]. Similarly, Kandeel et al. [32], in comparing previous seasons, found a higher rate in *Influenza* virus infection than RSV, although the latter caused the most severe cases. Notably, they observed a significant proportion of patients with RSV infection who missed their first exposure. Furthermore, when comparing previous seasons, Campo and Redlberger-Fritz noted an increased number of *Influenza* C virus cases in Austria during 2022, and they concluded that this could likely be attributed to a decreased prevalence in prior seasons due to non-pharmaceutical interventions (NPIs) [33]. 

**Figure 3:**
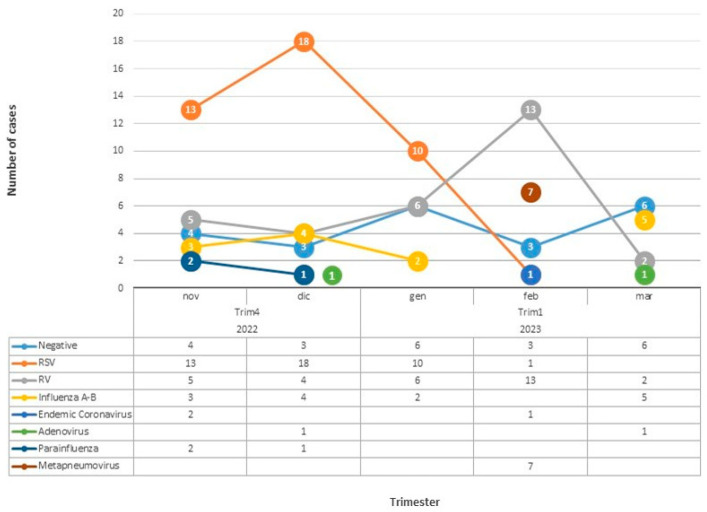
Distribution of viruses through the wave 2022–2023.

In our comprehensive epidemiological surveillance study focusing on respiratory viruses in children, we made several significant observations that shed light on the dynamics of viral infections during the studied periods. Our findings revealed a notable increase in the prevalence in respiratory viruses this year compared to previous years. Specifically, while 74 patients (53.26%) tested negative for viruses in 2021–2022, that figure decreased to only 22 patients (14.86) (*p* < 0.05) in 2022–2023. This discrepancy suggested a significant shift in viral activity within the population after lockdown. The lack of immune stimulation in children and adults could explain it. During 2022–2023, RSV emerged as the most prevalent virus (28.38%), confirming the trend already observed in the previous year (*p* > 0.05). In the previous year, RSV emerged as the predominant respiratory virus (23.2% of patients). In the current year, we witnessed a drastic and noteworthy surge in the circulation of other respiratory viruses. Compared to the previous year, a significantly higher prevalence in other respiratory viruses was observed (*p* < 0.05): RV, *Influenza* A and B, *Adenovirus*, and *Metapneumovirus*. In contrast, no cases of *Bocavirus* were reported, which showed a statistically significant difference from the previous year (*p* < 0.05). RSV, endemic *Coronaviruses*, and *Parainfluenza* viruses showed no statistical differences (*p* > 0.05). Another intriguing finding from our study was the observed peak incidence in RSV, which traditionally occurred around February before the pandemic. However, post-pandemic, the peak incidence appeared to shift to November. The changes in the seasonal pattern of RSV raised questions about the underlying factors influencing its circulation and highlighted the need for further investigation. Notably, in the previous epidemic, RSV exhibited dominance throughout the entire epidemic phase. However, an intriguing phenomenon unfolded this year, possibly attributable to viral competition. As shown in Figure 3, there was an abrupt disappearance in RSV in January, accompanied by a sudden and substantial increase in the prevalence in RV. These data marked a notable shift in the viral landscape, as RV became the most prevalent respiratory virus during January and February in our center. 

Several respiratory viruses can circulate during the same period and can simultaneously or sequentially infect the respiratory tract, leading to virus–virus interactions. At the host level, the course of a virus infection could be influenced by a previous or concurrent infection by another virus. Infection by a first virus could potentiate or decrease the infection and replication of a second virus, resulting in a positive (additive or synergistic) or negative (antagonistic) interaction [34]. Outside RV and RSV, all other viruses’ epidemic curves have remained stable for the whole epidemic season. The metapneumovirus emerged suddenly in February and had not been detected before. In 2022, similar results were reported by an Italian multicenter study, which indicated that the hospitalization curve for RSV bronchiolitis occurred earlier than in the pre-pandemic era [20]. This contrasting trend compared to the incidence in SARS-CoV-2 suggested the hypothesis that SARS-CoV-2 might exert competitive pressure on other respiratory viruses. Consequently, more susceptible individuals are more likely to be infected with viral infections due to reduced exposure, limited immunity duration, and reduced transplacental transfer of antibodies [35]. Our study uncovered a crucial finding regarding the current epidemic (2022/2023) compared to the previous one (2021–2022). While RSV was the predominant virus during the prior epidemic, a significant change occurred, with other respiratory viruses exhibiting an alarming surge in circulation. The reasons behind this shift remain elusive, warranting further investigation. The increased prevalence in other viruses, including but not limited to RV, indicates a substantial contribution to respiratory illness among children. These data in the viral landscape suggest a noteworthy change, where RSV, once the dominant player, has been replaced by other viral strains. The rise in RV as a primary culprit in pediatric respiratory infections is particularly noteworthy. Regarding the severity of respiratory infections, our study did not reveal any significant differences between the current and the previous epidemic wave. These data suggest that the overall severity of infections remained relatively stable despite the increased circulation of respiratory viruses. However, it is essential to continue monitoring and assessing the impact of these viral changes on disease severity and clinical outcomes. By comparing the two epidemics, the severity results were similar: the mean duration of hospitalization was 7.63 ± SD 4.07 in 2021–2022 and 7.47 days ± SD 3.54 in 2022–2023 (*p* = 0.73). Regarding the CRS, disease severity differences were not significant (*p* = 0.49). These findings confirm the crucial role of robust primary prevention strategies in counteracting respiratory infections in children. The need for effective measures, such as vaccination programs, hygiene practices, and public health interventions, becomes even more pronounced in light of the evolving viral epidemiology. Moreover, it is vital to emphasize the significance of ongoing epidemiological surveillance in the post-pandemic era. The shifts observed in viral prevalence and the patterns of respiratory infections highlight the dynamic nature of viral circulation and the need for vigilant monitoring. Understanding and replying to these epidemiological changes are essential to improve public health strategies, allocate resources effectively, and develop targeted interventions for adequate disease prevention and control.

## 5. Conclusions

Our comprehensive epidemiological surveillance study highlighted several noteworthy observations about viral dynamics during the investigated periods. In 2022–2023, a remarkable increase in the prevalence in respiratory viruses compared to previous years was reported, suggesting a significant change in viral activity within the population, possibly due to decreased immune stimulation. RSV emerged as the most prevalent virus during the 2022–2023 period, confirming the trend observed in the previous year. Additionally, there was a significant surge in the circulation of other respiratory viruses, including human RV, *Influenza* A and B, *Adenovirus*, and *Metapneumovirus*, compared to the previous year. We highlighted an intriguing phenomenon that occurred this year, potentially attributed to viral competition: in January, RSV abruptly disappeared and RV prevalence substantially increased, making RV the most prevalent respiratory virus during January and February. Despite the increased circulation of respiratory viruses, our study did not reveal significant differences in the severity of respiratory infections between the current and previous epidemics. These data suggest that the overall severity of infections remained relatively unchanged. These findings underscore the importance of robust primary prevention strategies in counteracting respiratory infections in children. Vaccination programs, hygiene practices, and public health interventions are crucial, especially in light of the evolving viral epidemiology. The observed shifts in viral prevalence and infection patterns highlight the dynamic nature of viral circulation and the need for vigilant monitoring. 

## Data Availability

The data presented in this study are available on request from the corresponding author. The data are not publicly available due to privacy restrictions.

## References

[1] Jefferson T., Del Mar C.B., Dooley L., Ferroni E., Al-Ansary L.A., Bawazeer G.A., Van Driel M.L., Jones M.A., Thorning S., Beller E.M. (2020). Physical interventions to interrupt or reduce the spread of respiratory viruses. Cochrane Database Syst. Rev..

[2] Zhang A., Surette M.D., Schwartz K.L., Brooks J.I., Bowdish D.M., Mahdavi R., Manuel D.G., Talarico R., Daneman N., Shurgold J. (2022). The Collapse of Infectious Disease Diagnoses Commonly Due to Communicable Respiratory Pathogens During the Coronavirus Disease 2019 Pandemic: A Time Series and Hierarchical Clustering Analysis. Open Forum Infect Dis..

[3] Curatola A., Lazzareschi I., Bersani G., Covino M., Gatto A., Chiaretti A. (2021). Impact of COVID-19 outbreak in acute bronchiolitis: Lesson from a tertiary Italian Emergency Department. Pediatr. Pulmonol..

[4] Ghirardo S., Ullmann N., Ciofi Degli Atti M.L., Raponi M., Cutrera R. (2021). Delayed season’s onset and reduction of incidence of bronchiolitis during COVID-19 pandemic. Pediatr. Pulmonol..

[5] Gastaldi A., Donà D., Barbieri E., Giaquinto C., Bont L.J., Baraldi E. (2021). COVID-19 Lesson for Respiratory Syncytial Virus (RSV): Hygiene Works. Children.

[6] Lazzerini M., Barbi E., Apicella A., Marchetti F., Cardinale F., Trobia G. (2020). Delayed access or provision of care in Italy resulting from fear of COVID-19. Lancet Child Adolesc. Health.

[7] Chiapinotto S., Sarria E.E., Mocelin H.T., Lima J.A.B., Mattiello R., Fischer G.B. (2021). Impact of non-pharmacological initiatives for COVID-19 on hospital admissions due to pediatric acute respiratory illnesses. Paediatr. Respir. Rev..

[8] Friedrich F., Ongaratto R., Scotta M.C., Veras T.N., Stein R.T., Lumertz M.S., Jones M.H., Comaru T., Pinto L.A. (2021). Early Impact of Social Distancing in Response to Coronavirus Disease 2019 on Hospitalizations for Acute Bronchiolitis in Infants in Brazil. Clin. Infect. Dis. Off. Publ. Infect. Dis. Soc. Am..

[9] Tempia S., Walaza S., Bhiman J.N., McMorrow M.L., Moyes J., Mkhencele T., Meiring S., Quan V., Bishop K., McAnerney J.M. (2021). Decline of influenza and respiratory syncytial virus detection in facility-based surveillance during the COVID-19 pandemic, South Africa, January to October 2020. Euro Surveill. Bull. Eur. Sur Les Mal. Transm.=Eur. Commun. Dis. Bull..

[10] Dolores A., Stephanie G., Mercedes S.N.J., Érica G., Mistchenko A.S., Mariana V. (2022). RSV reemergence in Argentina since the SARS-CoV-2 pandemic. J. Clin. Virol..

[11] Reina J., Arcay R.M., Busquets M., Machado H. (2021). Impact of hygienic and social distancing measures against SARS-CoV-2 on respiratory infections caused by other viruses. Rev. Esp. Quimioter. Publ. Of. Soc. Esp. Quimioter..

[12] Park K.Y., Seo S., Han J., Park J.Y. (2021). Respiratory virus surveillance in Canada during the COVID-19 pandemic: An epidemiological analysis of the effectiveness of pandemic-related public health measures in reducing seasonal respiratory viruses test positivity. PLoS ONE.

[13] Manti S., Giallongo A., Parisi G.F., Papale M., Presti S., Bianco M.L., Spicuzza L., Leonardi S. (2022). Impact of COVID-19 Pandemic and Lockdown on the Epidemiology of RSV-Mediated Bronchiolitis: Experience from Our Centre. Children.

[14] Guedj R., Lorrot M., Lecarpentier T., Leger P.L., Corvol H., Carbajal R. (2021). Infant bronchiolitis dramatically reduced during the second French COVID-19 outbreak. Acta Paediatr..

[15] Van Brusselen D., De Troeyer K., Ter Haar E., Vander Auwera A., Poschet K., Van Nuijs S., Bael A., Stobbelaar K., Verhulst S., Van Herendael B. (2021). Bronchiolitis in COVID-19 times: A nearly absent disease?. Eur. J. Pediatr..

[16] Camporesi A., Morello R., Ferro V., Pierantoni L., Rocca A., Lanari M., Trobia G.L., Sciacca T., Bellinvia A.G., De Ferrari A. (2022). Epidemiology, Microbiology and Severity of Bronchiolitis in the First Post-Lockdown Cold Season in Three Different Geographical Areas in Italy: A Prospective, Observational Study. Children.

[17] Van Summeren J., Meijer A., Aspelund G., Casalegno J.S., Erna G., Hoang U., Lina B., de Lusignan S., Teirlinck A.C., Thors V. (2021). Low levels of respiratory syncytial virus activity in Europe during the 2020/21 season: What can we expect in the coming summer and autumn/winter?. Euro Surveill. Bull. Eur. Sur Les Mal. Transm.=Eur. Commun. Dis. Bull..

[18] Chow E.J., Uyeki T.M., Chu H.Y. (2023). The effects of the COVID-19 pandemic on community respiratory virus activity. Nat. Rev. Microbiol..

[19] Presti S., Lo Bianco M., Mollica F., Manti S., Papale M., Parisi G.F., Leonardi S. (2022). Post-lockdown respiratory virus epidemiology: A monocentric observational study. Pediatr. Respir J..

[20] Nenna R., Matera L., Licari A., Manti S., Di Bella G., Pierangeli A., Palamara A.T., Nosetti L., Leonardi S., Marseglia G.L. (2022). An Italian Multicenter Study on the Epidemiology of Respiratory Syncytial Virus During SARS-CoV-2 Pandemic in Hospitalized Children. Front. Pediatr..

[21] Nayani K., Naeem R., Munir O., Naseer N., Feroze A., Brown N., Mian A.I. (2018). The clinical respiratory score predicts paediatric critical care disposition in children with respiratory distress presenting to the emergency department. BMC Pediatr..

[22] World Medical Association (2013). World Medical Association Declaration of Helsinki: Ethical principles for medical research involving human subjects. J. Am. Med. Assoc..

[23] Manti S., Licari A. (2018). How to obtain informed consent for research. Breathe.

[24] Teirlinck A.C., Johannesen C.K., Broberg E.K., Penttinen P., Campbell H., Nair H., Reeves R.M., Bøås H., Brytting M., Cai W. (2023). New perspectives on respiratory syncytial virus surveillance at the national level: Lessons from the COVID-19 pandemic. Eur. Respir J..

[25] Brealey J.C., Sly P.D., Young P.R., Chappell K.J. (2015). Viral bacterial co-infection of the respiratory tract during early childhood. FEMS Microbiol. Lett..

[26] Cohen R., Ashman M., Taha M.K., Varon E., Angoulvant F., Levy C., Ryback A., Ouldali N., Guiso N., Grimprel E. (2021). Pediatric Infectious Disease Group (GPIP) position paper on the immune debt of the COVID-19 pandemic in childhood, how can we fill the immunity gap?. Infect. Dis. Now.

[27] Fourgeaud J., Toubiana J., Chappuy H., Delacourt C., Moulin F., Parize P., Scemla A., Abid H., Leruez-Ville M., Frange P. (2021). Impact of public health measures on the post-COVID-19 respiratory syncytial virus epidemics in France. Eur. J. Clin. Microbiol. Infect. Dis. Off. Publ. Eur. Soc. Clin. Microbiol..

[28] Torres A.R., Guiomar R.G., Verdasca N., Melo A., Rodrigues A.P., Rede Portuguesa de Laboratórios para o Diagnóstico da Gripe (2023). Resurgence of Respiratory Syncytial Virus in Children: An Out-of-Season Epidemic in Portugal. Acta Med. Port..

[29] Chuang Y.C., Lin K.P., Wang L.A., Yeh T.K., Liu P.Y. (2023). The Impact of the COVID-19 Pandemic on Respiratory Syncytial Virus Infection: A Narrative Review. Infect. Drug Resist..

[30] Rao S., Armistead I., Messacar K., Alden N.B., Schmoll E., Austin E., Dominguez S.R. (2023). Shifting Epidemiology and Severity of Respiratory Syncytial Virus in Children During the COVID-19 Pandemic. JAMA Pediatr..

[31] Temte J.L., Goss M., Bell C., Barlow S., Temte E., Bateman A., Uzicanin A. (2023). Changing pattern of respiratory virus detections among school-aged children in a small community—Dane County, Wisconsin, September to December 2022. Influenza Other Respir. Viruses.

[32] Kandeel A., Fahim M., Deghedy O., Roshdy W.H., Khalifa M.K., El Shesheny R., Kandeil A., Naguib A., Afifi S., Mohsen A. (2023). Resurgence of influenza and respiratory syncytial virus in Egypt following two years of decline during the COVID-19 pandemic: Outpatient clinic survey of infants and children, October 2022. BMC Public Health.

[33] Camp J.V., Redlberger-Fritz M. (2023). Increased cases of influenza C virus in children and adults in Austria, 2022. J. Med. Virol..

[34] Piret J., Boivin G. (2022). Viral Interference between Respiratory Viruses. Emerg. Infect. Dis..

[35] Manti S., Leonardi S., Rezaee F., Harford T.J., Perez M.K., Piedimonte G. (2022). Effects of Vertical Transmission of Respiratory Viruses to the Offspring. Front. Immunol..

